# Monitoring and Promoting Physical Activity and Physical Fitness in All Age Groups

**DOI:** 10.1155/2024/9835396

**Published:** 2024-04-03

**Authors:** Bojan Masanovic, Stevo Popovic, Juel Jarani

**Affiliations:** ^1^Faculty for Sport and Physical Education, University of Montenegro, Niksic, Montenegro; ^2^Western Balkan Sport Innovation Lab, Podgorica, Montenegro; ^3^Faculty of Movement Sciences, Sports University of Tirana, Tirana, Albania

## 1. Introduction

Modern lifestyle brings with it many different challenges, no less than in the past, but modern challenges are significantly different. Therefore, it is very important to understand most of the challenges that modern man faces. Among other things, one of the most important challenges is the preservation of health. Health was one of the most important resources in the past, and it is just as important today. However, the factors influencing the impairment of good health have changed over time. In order for human being to maintain good health, it had to define the determinants of health and carefully monitor changes in their impact on health status over time. The modern lifestyle has changed dramatically [1, 2], and physical activity and physical fitness have become one of the very important determinants of the modern men's health [3]. In the past, these determinants were not the focus of scientists' attention because physical activity and physical fitness were taken for granted. However, the lifestyle in the last few decades has led to the physical inactivity of modern man [4, 5], which was recognized by scientists who established in their research that physical activity and physical fitness are associated with health benefits for individuals of all ages. Thus, it is now widely known that achieving a sufficient level of physical activity and physical fitness additionally contribute to better health-related biomarkers [6]. Therefore, if time is spent in sedentary behavior, it is realistic to expect negative health outcomes. Understanding and developing strategies to promote physical activity behavior is much more important than in the past, as it is essential to improve physical fitness levels [7]. Although, at the beginning, special attention was focused on children, later these strategies were also focused on other generations, primarily young people, adults, and also the elderly. There is a large number of studies that increasingly confirm that negative outcomes are visible in individuals of all ages [8–10]. Facing modern challenges and the desire to improve the health status of all age groups, this research topic was created, with the intention of helping the upbringing of monitoring and promoting physical activity and physical fitness in all age groups.

## 2. Contribution to the Field

The purpose of this research topic was to gather the latest knowledge in the field of monitoring and promoting physical activity and physical fitness in all age groups. The eight studies that emerged as the output of this special issue have advanced the field in several ways.

First, some very interesting findings were reached in this research topic related to the monitoring of trends in morphological characteristics among children. The authors of this study have examined the current state, dynamics, and direction of changes in morphological characteristics, over a 30-year period in Serbian children and adolescents among 7- and 11-year-old, and observed significant increase in height, body mass, and BMI in 7-year-old children from 1990 to 2020 [11]. On the other hand, Han et al. [12] have confirmed the effectiveness of a family-based intervention that integrated the family and preschool based on a smartphone app they created to improve the moderate-to-vigorous physical activity and physical fitness of preschool children during COVID-19.

Another important stream of work is reflected in the studies that analyzed: (1) traditional Chinese exercise (qigong) for chronic obstructive pulmonary disease [13] and (2) exercise rehabilitation among cancer patients [14]. These studies help us understand the therapeutic properties of exercise. Specifically, the authors of the first mentioned study have conducted systematic review and meta-analyses and reached scientific evidences that can help for the management of chronic obstructive pulmonary disease, while the second group of authors has conducted a bibliometric and visualized knowledge graph analysis and reached the research hotspots and frontiers of exercise rehabilitation among cancer patients via CiteSpace.

One more important stream of work is reflected in three studies that analyzed the physical performance of sportsmen. Akpinar [15] had investigated the motor lateralization profiles of youth soccer players and compared the same lateralization to nonathletes and reached very interesting outcomes that participation in soccer training improves lower limb coordination and decreases motor lateralization. Furthermore, Türkmen and Biçer [16] have examined the effects of an 8-week orienteering training on physical fitness parameters in adolescents and confirmed that orienteering training once a week for eight weeks resulted in positive developments in physical fitness parameters. Zheng et al. [17] have followed the Cochrane Collaboration guidelines and assessed the effect of short- and long-term detraining on trained individuals' V̇O2max through a systematic review and meta-analysis and reached interesting scientific evidences that are reflected in the fact that subjects with a higher V̇O2max training status have a greater decline in oxygen uptake after long-term training cessation.

Lastly, de Souza et al. [18] have evaluated ultramarathons with distances above 180 km in relation to runners' peak ages and performances and reached interesting data. They monitored the period between 2010 and 2020 and observed the following: (1) increase in the number of ultramarathon running events; (2) Europe had the highest number; (3) women had low participation; and (4) performance progression fell.

## 3. Conclusion

This special issue of the journal Biomedical Research International entitled “Monitoring and Promoting Physical Activity and Physical Fitness in All Age Groups” was created, with the special intention of helping the upbringing of monitoring and promoting physical activity and physical fitness in all age groups. It is very difficult to conclude whether the goal of this research topic was achieved or not. The subject was set very broadly, and any advance is satisfactory. Namely, although expectations were higher, it is the fact this research topic justified its existence. Eight high-quality studies have been collected that have advanced the field of monitoring and promoting physical activity and physical fitness in all age groups. To this end, this research topic leveraged high-quality research studying changes in all generations, from children to elderly, to offer guidance to scientists in the first place, and also to practitioners and policy makers around the globe on how to monitor and promote physical activity and physical fitness as well as alleviate the consequences of physical inactivity.

*Bojan Masanovic* *Stevo Popovic* *Juel Jarani*
